# Economic Support to Patients in HIV and TB Grants in Rounds 7 and 10 from the Global Fund to Fight AIDS, Tuberculosis and Malaria

**DOI:** 10.1371/journal.pone.0086225

**Published:** 2014-01-28

**Authors:** Linda M. Richter, Knut Lönnroth, Chris Desmond, Robin Jackson, Ernesto Jaramillo, Diana Weil

**Affiliations:** 1 HIV, AIDS, STIs and TB, Human Sciences Research Council, Durban, KwaZulu-Natal, South Africa; 2 Developmental Pathways for Health Research Unit, University of the Witwatersrand, Johannesburg, Gauteng, South Africa; 3 HIV/AIDS, TB, Malaria and Neglected Tropical Diseases (HTM), World Health Organization, Geneva, Switzerland; 4 World Food Programme, Rome, Italy; Public Health Agency of Barcelona, Spain

## Abstract

People with TB and/or HIV frequently experience severe economic barriers to health care, including out-of-pocket expenses related to diagnosis and treatment, as well as indirect costs due to loss of income. These barriers can both aggravate economic hardship and prevent or delay diagnosis, treatment and successful outcome, leading to increased transmission, morbidity and mortality. WHO, UNAIDS and the ILO argue that economic support of various kinds is essential to enable vulnerable people to protect themselves from infection, avoid delayed diagnosis and treatment, overcome barriers to adherence, and avert destitution. This paper analyses successful country proposals to the Global Fund to Fight AIDS, Tuberculosis and Malaria that include economic support in Rounds 7 and 10; 36 and 20 HIV and TB grants in Round 7 and 32 and 26, respectively, in Round 10. Of these, up to 84 percent included direct or indirect economic support for beneficiaries, although the amount constituted a very small proportion of the total grant. In TB grants, the objectives of economic support were generally clearly stated, and focused on mechanisms to improve treatment uptake and adherence, and the case was most clearly made for MDR-TB patients. In HIV grants, the objectives were much broader in scope, including mitigation of adverse economic and social effects of HIV and its treatment on both patients and families. The analysis shows that economic support is on the radar for countries developing Global Fund proposals, and a wide range of economic support activities are in place. In order to move forward in this area, the wealth of country experience that exists needs to be collated, assessed and disseminated. In addition to trials, operational research and programme evaluations, more precise guidance to countries is needed to inform evidence-based decision about activities that are cost-effective, affordable and feasible.

## Introduction

Economic support for patients serves a dual purpose: to help overcome economic barriers to use of health services [1], [2], [3], and to mitigate the financial burden of illness and care that can precipitate or worsen poverty [4], [5].

People with TB and/or HIV often experience severe economic barriers to health care in the face of high direct medical costs (consultations, drugs, diagnostics, hospitalization), as well as costs associated with transport, accommodation, food, substitute care, accompaniment and loss of income [6].

Most people who eventually start TB treatment manage to complete the treatment, but many do so at a very high price. A large proportion of patients end up in desperate financial situations as a consequence of both their inability to work due to illness, as well as direct and indirect costs of care and catastrophic borrowing to pay for care. The average total direct and indirect cost is often 10% or more [7], [8], [9] and can be as high as more than 100% of the annual household income [10]. Cost as a percentage of income is highest in poorer households [11], [12]. People with multidrug resistant tuberculosis, face even higher costs than those with drug-susceptible TB, due to longer and more complicated diagnosis and treatment, as well as more severe health conditions [13], [14].

Poor geographical and financial access to health services often prevent or delay health seeking among people with TB, especially the poorest [15]. Moreover, high direct and indirect cost of care constitute important determinants of poor treatment adherence, contributing to low cure rates and high risk of death among poor and vulnerable groups [16], [17]. While there are just a few trials on the impact of economic support on TB detection or treatment adherence, there is some evidence that such interventions, in combination with nutritional support, may improve MDR-TB treatment outcomes [18], [19], [20]. There is also evidence from settings that financial enablers or incentives can help improve uptake and adherence to treatment for latent TB [21], [22], [23].

Similarly, out-of-pocket expenses for the costs of treatment [24], as well as for transport and accommodation are known barriers for poor people to access HIV treatment and care [25], [26], [27], [28], even in middle-income countries [29]. These costs have been shown to affect the uptake of ARV treatment in Malawi [30] and to negatively impact ART adherence in Botswana, Brazil (both prior to free treatment), and Cameroon [31], [32], [33]. Attrition as a result of loss to follow-up is high in low and middle income countries [34], and fees for services (including for monitoring) and transport costs are related to lower retention [35]. Financial factors are also cited in studies of follow-up in prevention-of-mother-to-child-transmission programs [36]. Cost for patients of HIV treatment is estimated to correspond to 100% or more of annual income in China, Cote d'Ivoire, Indonesia, South Africa, Tanzania and Thailand [37], [38], [39]. Although there is little published research on economic support and treatment, improved uptake of HIV testing and treatment and improved treatment outcomes have been reported in respect of a number of social protection interventions, such as cash transfers [40], and food support [41].

Delayed, interrupted and incomplete treatment, in this case of both HIV and TB, not only poses a serious risk to individual health, but also increases the disease risk to others in the household and beyond [21]. Moreover, catastrophic cost of illness in itself increases vulnerability of household members. For poor households, a cost burden around 10% of annual income for medical care is calculated to lead to cuts in consumption, sale of assets, and debt that is likely to result in further impoverishment with the threat of destitution [39]. Household strategies to manage out-of-pocket medical costs threaten their future health and wellbeing. For example, diminished food intake, through subsequent malnutrition, can increase the risk of TB disease amongst those infected [42], [43].

WHO [44], UNAIDS [45], ILO [46] and others argue strongly that transfers and additional forms of social protection are essential to enable vulnerable people to protect themselves from infection, increase access to diagnosis and treatment, improve adherence to treatment, and prevent destitution. However, the extent of inclusion of such interventions in disease programmes, such as those for TB and HIV, is poorly documented. Several programs - including some financed by the Global Fund to Fight AIDS, Tuberculosis and Malaria – are providing economic support in a variety of forms, from cash transfers for poverty alleviation to transport reimbursement and meals provided to enable and incentivise attendance at health facilities for care.

In this paper, we analyse Rounds 7 and 10 HIV and TB grants from the Global Fund to Fight AIDS, Tuberculosis and Malaria (the Global Fund). We identify funded programs and describe the stated rationale for, and extent and nature of these efforts. For those with sufficient information, we calculate the number of individuals benefitting from economic support, the proportion of the total budget and the annual US$ per person benefit.

## Methods

Two rounds of country proposals to the Global Fund for HIV and TB grants approved by the Technical Review Panel for funding were examined to determine if they include economic support to address barriers to prevention, treatment and care and support. Proposals submitted for HIV and TB support in Round 10 (year 2010) provide an indication of recognition within country, in recent years, of the perceived importance of providing economic support to success of their disease programme, but do not indicate the actual amount allocated through grant negotiations to economic support, nor achieved expenditure in the first two years of the grant. For this reason, Round 7 TB and HIV grants (year 2007) were also examined to determine not only what was included in proposals, but also how much of the budget for economic support was spent at the end of the first phase of the grant. The emphasis in this analysis is on Round 10 (tables included), with Round 7 included for comparison (suggested to be included as web material).

From an initial review of grant documents in Rounds 7 and 10, we distinguished three forms of economic support that could be reliably coded.

1. *Direct Economic Assistance*. These are *direct transfers of money*, such as cash paid as part of a social security system or a program incentive, transport reimbursements, treatment allowances, and the like that are paid directly to affected individuals.
2. *Indirect Economic Assistance.* These are *indirect transfers* through, for example, food parcels, food or travel vouchers, and payment of health insurance for individuals, households or families. This indirect assistance provides some relief to the household for necessary expenditure on these items, and thus may free up resources for other categories of household consumption. Some forms of indirect assistance may also be converted into cash, even if inefficiently; for example, food parcel items may be sold to pay for transport.
3. *Enterprise Assistance*. This includes training programmes or microcredit that aim to assist individuals or families to generate income. However, the benefits which accrue to the individual or household are not quantifiable at the time of award because the realization of economic returns is dependent on factors beyond the intervention and occurs at a later point in time.

Reimbursements or payments for peer counsellors or ‘expert patients’ [49] were excluded, as were incentives paid to health service staff. Legal aid, advocacy, patient charters and other activities that could not, on the face of it, be readily translated into an economic benefit for an individual or household were also excluded.

For Round 10, the proposal narrative and the Year 1 budget was analysed and, where possible, converted to US$ dollar per person per annum direct or indirect benefit. For Round 7, the original proposal, the program grant agreement, the grant performance report and the grant score card were analysed to determine activities and budget allocations at the end of the second year of implementation of the grant.

There were challenges to coding in all narratives assessed: when it was not clear if the same or different individuals are covered in a time period, how activities are spread over time;how units (for example, nutrition supplements) are allocated to individuals, and whether all operational details of providing economic support were included in the proposalWhen a budget line contained activities that qualify as a form of economic support, together with activities that do not, we split the budget equally between the activities as has been done in previous analyses of Global Fund grants [50]. Where budgets were submitted in Euros, conversion to US dollars was made at the exchange rate pertaining on 20 August 2010, the deadline for submission, in the case of Round 10 proposals ($1.28), and on 4 July 2007 for Round 7 ($1.36).

Proposals were also analysed with respect to: 1) the stated rationale for the intervention (income assistance, income generation, promote treatment adherence, poverty relief etc); 2) to whom the assistance is targeted (an individual, household or specified group); 3) the monetary value of a unit of the assistance; 4) the number of individuals or households targeted; and 5) the proportion of the total grant budgeted for economic support.

All Global Fund documents are publicly accessible off the Global Fund home page (www.globalfund.org).

## Results

The results from Round 10 are presented Tables 1 and 2 for HIV and TB grants, respectively. Results from Round 7 are available in support material on the web (Tables S1 and S2). Table 3 summarises the proportion of direct, indirect and enterprise economic assistance in Rounds 7 and 10 for HIV and TB.

**Table 1:** Round 10 HIV proposals that included direct and indirect forms of economic support (Year 1).

Country	Type of support	Description	Rationale	Target group	No of people, units	Total Y1 budget ($US)	Budget PB/PA ($US)	Portion of total budget (%)
Burkina Faso	Indirect	Nutrition as treatment	Increase adherence and effectiveness of treatment	PLWHA	3 223	57 660	18	0.2
Burkina Faso	Indirect	Health costs	Reduce economic impact of HIV on PLWHA and their families	OVC	1 500	22 867	15	0.2
Burkina Faso	Indirect	Nutrition support	Reduce economic impact of HIV on PLWHA and their families	OVC, Families	1 500	45 735	30	0.3
Burkina Faso	Indirect	School and training support	Reduce economic impact of HIV on PLWHA and their families	OVC	23 000	670 776	29	3.4
Cameroon	Indirect	Food support in PMTCT	-	Children	6 917	80 670	12	0.6
Cameroon	Indirect	Nutrition support	Reduce negative economic impact, equitable access	OVC	20 000	194 569	10	1
Cameroon	Indirect	Education support	Reduce negative economic impact, equitable access	OVC	20 000	389 120	19	2
Eritrea	Indirect	Nutrition as treatment	Improve adherence, reduce burden of care	PLWHA	3 500	525 000	150	8
Eritrea	Enterprise	Vocational training	Reduce HIV transmission & sex worker vulnerability	SW	150	216 750	1 445	3
Guinea	Indirect	Food supplement	-	CLWHA	200	51 318	256	2
Guinea	Indirect	Therapeutic nutrition	-	PLWHA	236	14 300	50	0.3
Guinea	Indirect	Food support	-	PLWHA	200	10 000	50	0.2
Guinea	Indirect	School assistance	Prevention, as part of holistic treatment	OVC	1000	50 000	50	1
Guinea	Enterprise	Income generating activities	Prevention, as part of holistic treatment	PLWHA	100	100 000	1 000	1.6
Guinea	Enterprise	Job retraining	Prevention, as part of holistic treatment	PLWHA	200	12 000	50	0.2
Kenya	Indirect	Nutrition as treatment	Enhance treatment effectiveness. Survival support	PLWHA	137 300	811 720	23	0.2
Kenya	Indirect	Nutrition as treatment	Enhance treatment effectiveness. Survival support	PLWHA	65 900	480 912	27	0.1
Kenya	Indirect	OVC nutrition support	Reduce economic impact on affected groups	OVC	1 500	45 735	30	0.3
Kenya	Indirect	OVC education	Reduce economic impact on affected groups	OVC	23 000	670 733	36	3
Kyrgyzstan	Indirect	Food, hygiene packages	-	OVC	50	15 000	300	0.3
Kyrgyzstan	Indirect	Support package	-	PLWHA, Children	-	1 600	-	0.3
Kyrgyzstan	Indirect	Food packages	Motivation	IDUs, PLWHA	130	13 000	100	0.3
Kyrgyzstan	Indirect	Food packages	-	IDUs, PLWHA	-	6 000	-	0.1
Macedonia	Indirect	Provision of food	-	Homeless IDUs	-	125	-	0.01
Macedonia	Enterprise	IGA Creative worshops	-	IDUs	-	2 400	-	0.01
Macedonia	Enterprise	Vocational training	Allow MARPs to compete for job, re-integration	MARPS	20	5 120	200	0.3
Malaysia	Indirect	Food, transport	Incentive	IDUs	104	21 376	187	1
Malaysia	Indirect	Food, transport	Incentive	SWs	132	24 750	187	1
Morocco	Indirect	Solidarity food baskets	Quality treatment, preservation of human rights	PLWHA	67	25 093	375	0.4
Morocco	Indirect	Transport costs	Quality treatment, preservation of human rights	PLWHA	90	6 750	75	0.1
Morocco	Indirect	Transport costs	Quality treatment, preservation of human rights	CLWHA, Families	40	3 000	75	0.05
Morocco	Indirect	School supplies	Quality treatment, preservation of human rights	CLWHA	20	1 250	62	0.02
Morocco	Enterprise	Income Generating Activities	Quality treatment, preservation of human rights	PLWHA	-	-	-	-
Multi –Country Americas (REDCA)	Direct	Educational incentives	-	Transsexuals	3 720	167 400	46	8
Multicountry East Asia And Pacific (APN)	Indirect	Transport	Enable treatment access	PLWHA	100	2 100	21	0.1
APN+	Indirect	Nutrition, medicine support	Enable treatment access	PLWHA	100	3 600	36	0.1
APN+	Indirect	Transport	Enable treatment access	PLWHA	267	12 816	48	0.3
APN+	Indirect	University education	Strengthen capacity of APN+ network	PLWHA	10	151 500	15150	2.5
APN+	Indirect	Short courses	Strengthen capacity of APN+ network	PLWHA	40	80 000	2 000	1
Nepal	Direct	Food subsidy	Reduce vulnerability to HIV	FSWs	480	9 864	21	0.1
Nepal	Direct	Food subsidy	Reduce vulnerability to HIV	MSM	720	14 796	21	0.2
Nepal	Direct	Cash transfer	Reduce economic and social vulnerability	OVC, Families	100	6 164	62	0.6
Nepal	Enterprise	Livelihood training	Reduce economic and social vulnerability	OVC	100	13 500	135	0.3
Peru	Enterprise	Micro-enterprise, training	-	Transsexuals	-	13 734	-	0.5
Peru	Indirect	Secondary education grant	-	Transsexuals	80	57 600	360	1
Peru	Indirect	Technical degree grant	-	Transsexuals	40	200 000	170	4
Sao Tome	Indirect	School support orphans	Improve quality of life	OVC	40	14 400	360	2
Sao Tome	Indirect	Food supply for orphans	Improve quality of life and reduce mortal	OVC	40	5 760	144	4
Sao Tome	Indirect	Infant formula	Reduce child mortality	Children		30 209	20	6
Sudan (North)	Enterprise	Income Generating Activities	Empowerment, enabling environment	PLWHA	-	50 000	-	0.4
Syria	Enterprise	Income Generating Activities	Less vulnerable to sexual violence, infection	Sex Workers	-	6 250	-	0.1
Timor Leste	Direct	Transport assistance	-	MSM	180	360	2	0.01
Timor Leste	Direct	Transport assistance	-	SWs	204	408	2	0.01
Thailand	Direct	Grants for income generation	Increase household economic capacity, improve acceptance	OVC, Families	1 674	104 625	62	1.4
Thailand	Direct	Cash grants	Increase household economic capacity, improve acceptance	PLWHA, Families	335	15 703	187	1.4
Ukraine	Indirect	Transport, food, incentives	Support for patients	MSM, Children	2 000	2 099	1.05	-
Vietnam	Indirect	Educational support	Equal educational opportunities	Children, MARPS	103	11 000	100	0.0
Vietnam	Indirect	Nutritional support	-	PLWHA	900	108 000	120	0.5
Vietnam	Indirect	Nutritional support	-	PLWHA	50	500	10	<0.01
Zambia	Indirect	Nutritional supplements	Improve effectiveness of ART. Safety net.	CLWHA	2370	53 236	22	0.1
**TOTAL**					**333 129**	**5 613 753**	6	

APN+  =  Multi-country East Asia and Pacific; CLWHA  =  Children Living with HIV and AIDS; IDUs  =  Injecting Drug Users; MARPS  =  Most At Risk Populations; MSM  =  Men Who Have Sex With Men; OVC  =  Orphans and Vulnerable Children; PLWHA  =  People Living with HIV and AIDS; SW  =  Sex Workers.

**Table 2:** Round 10 TB proposals in Round 10 that included direct and indirect forms of economic support (Year 1).

Country	Type of support	Description	Rationale	Target group	No of people, units	Year 1 budget (US$)	Budget per beneficiary (US$)	Portion of total grant budget
Armenia	Direct and indirect	Nutrition package and transport allowance	Promote treatment adherence	Patients, families	240	122030	508	5.0%
Bangladesh	Direct and indirect	Food package, travel vouchers, and cash-transfer	Not clear	Patients	1845	228766	124	0.9%
Colombia	Indirect	Food package	Promote treatment adherence	Patients	1200	20000	17	0.7%
Djibouti	Indirect	Food package	Promote diagnosis, treatment adherence	Patients	5313	105960	20	6.0%
Eritrea	Indirect	Food package, transport voucher and detergents for MDR patients	Promote treatment adherence	Patients	20	10667	533	0.2%
Ghana	Not clear	Enablers, not specified	Promote treatment adherence	Patients	19000	493600	26	2.0%
Honduras	Indirect	Food package and transport support	Promote treatment adherence	Patients	240	69300	289	2.2%
Indonesia	Indirect	Food package	Not clear	Patients	NA	NA	NA	NA
Jordan	Direct and indirect	Food package and travel reimbursements	Promote treatment adherence	Patients	200	60000	300	10.0%
Lao PDR	Indirect	Food package	Promote treatment adherence	Patients	4034	168800	42	0.3%
Macedonia	Indirect	Food package and transport voucher	Promote treatment adherence	Patients	250	38400	154	3.0%
Mongolia	Direct, indirect and enterprise	Food package, food allowance and occupational training	Promote treatment adherence	Patients	110	78750	716	
Namibia	Indirect	Transport assistance for MDR-TB patients	Not clear	Patients	300	7500	25	0.1%
Niger	Indirect and Enterprise	Food package, income generating fund	Promote treatment adherence	Patients	5118	463438	91	10.1%
Russian Federation	Direct and indirect	Food package and transport reimbursements, MDR-TB patients	Promote treatment adherence	Patients	242570	2097294	10	8.0%
Senegal	Indirect	Food package for MDR-TB patients	Not clear	Patients	107	30852	288	0.5%
Somalia	Direct and indirect	Travel allowance and live stock to MDR-TB patients	Promote treatment adherence and poverty alleviation	Patients	88	29200	332	6.2%
Swaziland	Indirect	Food package and transport assistance for MDR-TB patients	Promote treatment adherence, poverty alleviation	Patients	375	93648	250	0.7%
Uganda	Direct and indirect	Food vouchers and transport refund for MDR-TB patients	Promote treatment adherence	Patients	200	165000	825	2.9%
**TOTAL**					**280970**	**4161175**	**17**	

**Table 3:** Direct, indirect and enterprise economic support activities in Rounds 7 and 10 grants for HIV and TB.

Economic Support	TB and HIV Rounds 7 and 10			
	TB Round 7 (21 activities)	TB Round 10 (38 activities)	HIV Round 7 (41 activities)	HIV Round 10 (59 activities)
Direct	3 (14%)	7 (18%)	6 (15%)	8 (14%)
Indirect	13 (62%)	27 (71%)	24 (59%)	41 (69%)
Enterprise	5 (24%)	3 (8%)	11 (27%)	10 (17%)

There were 32 and 26 approved HIV and TB proposals, respectively, including cross-cutting proposals in Round 10, of which 21 HIV (66%) and 19 (73%) TB proposals included either direct or indirect economic support for beneficiaries.

For HIV, it was estimated that more than 333 000 affected people would benefit from economic support in the first year of the five-year proposals. The estimated number of beneficiaries is probably an underestimate, as individuals could not always be tallied from activities targeted at groups or organizations. The total value of economic support was $US5.6 m.This is equivalent to an average $US17 per person per annum. The percentage of HIV grants budgeted for economic support ranged from less than 0.01% to 8%.

Approved TB grants from this round included benefits in the first year of the grant for close to 240 000 people with a value of US$4.3 m, equivalent to average US$15 per person per annum. The percentage of TB grants budgeted for economic support range from 0.1% to 10%.

In Round 7, 26 of 36 HIV proposals (72%) and 18 of 20 TB proposals (84%) included economic support for patients and other affected people (data in web material). The proportion of the proposed or actual grant budget allocated to direct, indirect or enterprise assistance was less than 1% in most grants in Round 7.

With respect to economic support as defined in this analysis, a higher proportion of TB as compared to HIV proposals included economic support. The language in TB documents was also more standardised than those for HIV. All TB proposals in both Rounds listed TB patients as target groups for economic support. Seven (39% of TB grants) targeted economic support only to multi-drug resistant TB, while the rest targeted both patients with drug-resistant and patients with drug-susceptible TB. There was also a consistent rationale for providing economic support TB grants. In most cases it was stated as encouraging initiation and adherence to treatment, and generally took the form of food or transport support as incentives or enablers. Only in a few cases was economic support also provided for either socioeconomic rehabilitation of treated TB patients or to lessen the economic burden of illness on families.

Economic support in HIV proposals has a wider scope and therefore less standardised terminology. In Round 10, 10 of the 21 approved HIV proposals targeted Persons Living with HIV or AIDS (PLWHA, adults and/or children), 9 targeted Orphans and Vulnerable Children (OVC) and 9 targeted Most at Risk Populations (MARPs) or key populations. In the latter category, 5 grants targeted sex workers, 3 targeted Men who have Sex with Men (MSM), 2 Injecting Drug Users (IDUs) and 2 transsexuals. In two proposals, the economic support was proposed for the children of key populations. A slightly different pattern was found in Round 7 grants, indicating that support for key populations increased from Round 7 to 10 in line with the Global Fund's 2009 strategy for Sexual Orientation and Gender Identity (SOGI); 19 of the 26 Round 7 grants targeted PLHWA, adults and/or children), 8 targeted OVC and only 2 grants (both from Afghanistan) targeted key populations and the children of key populations. Children and families are frequently targeted in HIV grants – of 58 instances of Round 10 transfers, 27 (47%) included children and families, and in Round 7, 71% included children and families. In contrast, no TB grants in Round 10 included children and families, and only 2 of 21 transfer types in Round 7.

Of 59 distinct economic support activities in HIV and TB grants in Round 10, close to half (n = 27) involved food and nutrition, 16 income generation and 8 education. A similar distribution of support was found in Round 7 proposals.

In TB grants, food and nutrition support was consistently related to treatment objectives, particularly adherence and treatment, whereas in HIV grants it was more frequently related to socioeconomic vulnerability associated with HIV and AIDS. Food and nutrition was seldom justified by treatment initiation, adherence or treatment outcomes in HIV grants.

Most economic support was indirect. The overall proportion of direct, indirect and enterprise economic support was the same across the two diseases, but TB and HIV grants differ with respect to the nature of direct and indirect economic support provided. In TB grants, direct support takes the form of cash for food and transport. In HIV grants it also includes cash transfers to households caring for vulnerable children. All indirect economic support for TB was for food and/or for transport. For HIV, in contrast, indirect support also included maternity benefits, further education for members of a network of HIV-positive people to strengthen its capacity, and health insurance for vulnerable children. In both HIV and TB grants, enterprise support consisted principally of vocational training, micro-enterprise and other forms of assistance aimed at self-sufficient livelihoods and income-generation.

## Discussion

This analysis indicates that many countries are aware of the economic burden on patients and include financial and material support in their Global Fund grants in an attempt to mitigate these effects. This was not skewed to a particular region; most countries in all regions that submitted Global Fund TB and HIV proposals in Rounds 7 and 10 requested some funds for economic support to patients. Although the amount allocated for such support constituted a very small part of the total grant budgets, and the budget per beneficiary was relatively small, these contributions are likely to be important for vulnerable patients and households. Economic support is likely to be especially appreciated when out-of-pocket expenses are needed to compensate for infrastructuraldeficiencies for which governments are responsible, such as long distances to health services and lack of transport.

Economic support in TB grants tends to be more standardised and linked more directly to patient treatment and adherence than in HIV grants. This trend can be explained by the guidance provided in the Global Fund TB and Human Rights Information Note, which explicitly comments on the value of economic support to overcome access barriers, whereas the HIV and Human Rights Information Note does not. In contrast, the HIV and Human Rights Guidance Note emphasises social exclusion, marginalization, criminalization, stigma and inequity as major barriers to service uptake (http://www.theglobalfund.org/en/accesstofunding/notes/). HIV proposals highlight more clearly than TB grants the risk of destitution associated with disease, caused by losses of livelihoods and income. This does not mean that social and economic factors do not feature prominently in the vulnerability to and consequences of TB [42]. However, in Global Fund grants there seems to be more emphasis on using economic support as a means to enhance access and adherence to TB treatment than for mitigation of catastrophic costs related to illness.

There is some previously published evidence that economic support in the form of enablers, incentives and reimbursements can improve TB and HIV service uptake and adherence to treatment [21], [22], [23], [40], [41], [47]. However, the data on what type of incentives and enablers are more effective and cost-effective is weak and inconsistent. Economic support as a means to compensate or mitigate catastrophic costs has direct effects on the household economic situation, as shown in other health areas [40], though opportunity costs and potential perverse effects need be further analyzed. The grantees of the Global Fund seem to be ideal programmes where such analysis could be conducted, with the ultimate purpose of contributing to build evidence for global policy update. For this, detailed in-country and in-programme evaluations are needed to assess the effectiveness of different forms of operationalization.

Most forms of economic support financed by the Global Fund are indirect. Experience in poverty alleviation and food security has demonstrated that cash transfers can be more economically efficient than in-kind transfers in many settings. Cash is preferred by beneficiaries because it gives them the freedom to pay for or buy what they most need [48]. More programs involving cash (conditional or non-conditional) plus other indirect transfers need to be analysed to determine which combinations of transfers are most effective for TB and HIV, for different components of programmes – prevention, treatment, mitigation – and in different settings.

## Conclusions

A large proportion of Global Fund grants for TB and HIV in Rounds 7 and 10 included an element of economic support, even though the amount constitutes a very small proportion of the total grant. The amounts allocated are not commensurate with the predicted financial burden of TB or HIV on affected households. This suggests that, while countries are aware of the added value of economic support in TB and HIV care and prevention, there remains a gap of considerable burden on poor patients. In TB grants, the objectives of economic support were generally consistently stated, and focused on mechanisms to improve treatment uptake and adherence, and the case is most clearly made for MDR-TB patients. In HIV grants, the objectives were much broader in scope, including mitigation of adverse economic and other social effects of HIV and its treatment. This probably reflects the different rationale for TB and HIV support. In the former, the aim is to achieve a cure for both individual and public benefit, whereas for HIV there are considerations of long-term welfare assistance.

This analysis could not examine critical concerns with respect to the operationalization, design, management, monitoring or evaluation processes pursued for these interventions. In addition, further information is needed on whether and the extent to which the funds were used for the proposed interventions. Lastly, the available documentation does not enable an assessment of what proportion of economic support interventions were created specifically under the grants and fully funded by the Global Fund, and what proportion is or could be sustained under larger national schemes, such as social protection mechanisms involving cash transfers, or food and transport subsidies.

Nonetheless, the review, the first of its kind with respect to funds for economic support for country programmes, demonstrates that economic support is on the radar. The is also increasing interest for countries developing Global Fund proposals, and a wide range of activities are already in place. However, the rationale and evidence base for the activities are rarely well-established, nor have variations in operational features been tested. In order to move forward in this area, the wealth of country experience that exists must be collated, assessed and disseminated. Randomised control trials are needed, as is operational research and programme evaluations to assess the need and optimal format for, and the impact of, economic support in TB and HIV prevention, treatment and care. More guidance for countries is needed to inform evidence-based decisions about activities that are cost-effective, affordable, feasible, sustainable and responsive to the expressed needs of the persons served.

## Supporting Information

Table S1 Round 7 HIV grants that included direct and indirect forms of economic support in Phase 1 (first two years).
(DOCX)

Table S2 Round 7 TB grants that included direct and indirect forms of economic support in Phase 1 (first two years).
(DOCX)
