# Supplementary material for: Economic Support to Patients in HIV and TB Grants in Rounds 7 and 10 from the Global Fund to Fight AIDS, Tuberculosis and Malaria
Source: PLoS One. 2014 Jan 28;9(1):e86225. doi: 10.1371/journal.pone.0086225 (PMC3904874; doi:10.1371/journal.pone.0086225)
Supplement: Table S1 — Round 7 HIV grants that included direct and indirect forms of economic support in Phase 1 (first two years). (DOCX) [file pone.0086225.s001.docx]

Table S1: Round 7 HIV grants that included direct and indirect forms of economic support in Phase 1 (first two years)

| **Region / Country** | **Type of transfer** | **Description** | **Rationale** | **Target group** | **No of people, units** | **Total expended on Living Support** | **$US/**  **pp/pa** | **Portion of total budget** |
| --- | --- | --- | --- | --- | --- | --- | --- | --- |
| **East Africa, Indian Ocean** |  |  |  |  |  |  |  |  |
| Congo  ZAE-708-G06-H | Indirect | Assistance with school fees | Ensure socioeconomic care of PLWHA and affected people | PLWHA OVC | 12 440 | - | - | - |
|  | Enterprise | Income generating activities | Reduce the dependence of affected people and their housheolds | PLWHA  OVC | - | - | - | - |
| Ethiopia  ETH-708-G07-H | Indirect | Supplementary food and nutrition support | Reduce vulnerability and mitigate impact on PLWHA and their families.  Malnourished children and adults | PLWHA  FAMILIES | 76 305  1 213 | $3 127 278 | $20  $1 289 | 3% |
|  | Direct | Cash to purchase food | Reduce vulnerability and mitigate impact on PLWHA and their families | PLWHA | 4 958 | As above | $315 |  |
|  | Enterprise | Vocational training in business skills | Sustainable source of livelihood | PLWHA | 8 673 | As above | $180 |  |
| ETH-708-G08-H | Indirect | Nutrition support, subsidised transport | Mitigate impact on PLWHA and their families | PLWHA  FAMILIES | 57 747 | - | - | - |
|  | Enterprise | Income generation activities | Create jobs for PLWHA and their families | PLWHA  FAMILIES | 8 000 | - | - | - |
|  | Direct | Cash for food, clothing, health etc to initiate IGA activities | Increase holistic support for OVCs and families and purchase food not included in food supplements | OVC  FAMILIES | 17 332 | - | - | - |
| ETH-708-G09-H | Indirect | Food, shelter, clothing, health service subsidies | Increase holistic support for OVCs and families | OVC  FAMILIES | 49 612 | 7 596 843 | $23 | 8% |
|  | Direct | Financial support | Increase holistic support for OVCs and families | OVC  FAMILIES | 122 456 | As above | $9 | - |
|  | Enterprise | IGA support | Increase holistic support for OVCs and families | OVC  FAMILIES | 7 574 | As above | $163 | - |
| Kenya  KEN-708-G09-H | Indirect | Nutrition supplementation,t therapeutic feeding for PLWHA (adults & children) | Scale up and sustain PLWHA on ART | PLWHA (Adults & Children) | 11 383 | - | - | - |
| KEN-708-G10-H | Indirect | Nutrition supplementation,t therapeutic feeding for PLWHA (adults & children) | Remove barriers to initiating ART | PLWHA (Adults & Children) | 19 556 | - | - | - |
| Rwanda  RWN-708-G09-H | Direct | Funding for IGA | Provide care and support for the chronically ill through PLWHA associations | PLWHA |  | $1 449 165 |  | 1% |
|  | Indirect | Minimum package of support, including nutrition | Improves the lives and livelihoods of those made vulnerable by HIV/AIDS | OVC | 79 950 | As above | $9 | - |
| Uganda  UGD-708-G07H | Indirect | Minimum package of support to facilities and patients | Reduce PMTCT | PLWHA | 4 266 | - | - | - |
|  |  |  |  |  |  |  |  |  |
| **Southern Africa** |  |  |  |  |  |  |  |  |
| Lesotho  LSO-708-G05-H | Indirect | Essential supplies (school uniforms, toiletries, food, shelter) | Provide support for OVC | OVC  Families | 83 903 | $2 972 388 | $17 | 9% |
|  | Direct | Financial support to attend high school | Empower OVC by providing education and training to ensure a health future | OVC | 14 316 | As above | $103 | - |
|  | Enterprise | Vocational training in business and entrepeneurship, Braille equipment for disabled children | Empower OVC by providing education and training to ensure a health future | OVC  Disability | 6 4 33 | As above | $231 | - |
| Malawi  MLW-708-G07-H | Enterprise | Vocational training | Reduce the vulnerability of young people to HIV, especially girls and women | Vulnerable youth | 640 | $819 513 | $640 | 2% |
|  | Direct | Microcredit | Support vulnerable youth with microcredit start-up kits | Vulnerable youth | 467 | As above | $877 |  |
| Swaziland  SWZ-708-G05-H | Indirect | Basic external support to households of chronically ill persons (education, food) | Improve the quality of life of OVC | OVC  Households | 356 802 | $2 400 000 | $3.30 | 3% |
| **West, Central Africa** |  |  |  |  |  |  |  |  |
| Central African Republic  CAF-708-G05-H | Indirect | Nutritional support | Improve the quality of life of PLWHA | PLWHA  (Adults & Children) | 5 959 | $10 028 | $0.80 | 0.02% |
| Guinea-Bissau  GNB-708-G05-H | Indirect | Nutrition and education support | Free basic external support for households caring for OVC | OVC  Families | 40 577 | $1 916 | $0.08 | 0.01% |
| **Latin America, Caribbean** |  |  |  |  |  |  |  |  |
| El Salvador  SLV-708-G05-H | Indirect | Nutritional support for PLWHA,  School supplies, shows, uniforms for CLWHA | Care and support for chronic patients and to send CLWHA to school | PLWHA (Adults & Children) | - | - | - | - |
| El Salvador  SLV-708-G06-H | Enterprise | Vocational training | Enable PLWHA to enter the workforce | PLWHA | 219 | - | - | - |
| Haiti  HTI-708-G06-H | Enterprise | Micro-credit | More efficient standardised HIV-related social service package | PLWHA | 303 | $220 068 | $363 | 1% |
|  | Enterprise | Vocational training | More efficient standardised HIV-related social service package | PLWHA | 2 210 | As above | $49 | - |
|  | Indirect | Maternity benefits programme | Maternity and infant care package for HIV+ women and their infants | PLWHA  Children | 1 762 | As above | $62 | - |
| Jamaica  JAM-708-G02-H | Indirect | Nutrition supplies | Increase access to treatment for CLWHA | CLWHA | 40 | $30 227 | $377 | 0.00% |
| REDCA+  MAR-708-G01-H | Indirect | Scholarships | Strengthening of civil society and institutional capacity building | PLWHA | 143 | $456 769 | $1 597 | 0.08% |
|  | Enterprise | Training in management and IGAs | Strengthening of civil society and institutional capacity building | PLWHA | 318 | As above | $718 | - |
| **Middle East, North Africa** |  |  |  |  |  |  |  |  |
| Niger  NGR-708-G08-H | Indirect | Social and nutritional support for households caring for OVC | Support OVC | OVC  Families | 7 926 | $872 915 | $55 | 0.03% |
| **Eastern Europe, Central Asia** |  |  |  |  |  |  |  |  |
| Kazakhastan  KAZ-708-G03-H | Indirect | Care and support services | Improve ARV adherence | PLWHA | 3 252 | - | - | - |
| Kosovo  KOS-708-G02-H | Indirect | Support packages, including food | Improve the quality of life of PLWHA by promoting a supportive environment | PLWHA | 292 | $5 343 | - | 0.1% |
| Kyrgyzstan  KGZ-708-G05-H | Indirect | Food packs to families and breast milk substitutes for babies <1yr born to HIV+ mothers | Preventing HIV transmission from parents to children | PLWHA  Children | - | $282 085 | - | 0.9% |
| Macedonia  MKD-708-G03-H | Indirect | Support to access treatment | Improve access to treatment | PLWHA | - | $46 348 | - | 0.7% |
| **South, West Asia** |  |  |  |  |  |  |  |  |
| Afghanistan  AFG-708-G04-H | Indirect | Provision of basic livelihood to children at risk and disabled people | Ensure access to prevention, treatment and care for most at risk and vulnerable populations | Children  MARPS  Disability |  | $276 114 |  | 0% |
| Afghanistan  AFG-708-G05-H | Indirect | Provision of basic livelihood to children at risk and disabled people | Ensure access to prevention, treatment and care for most at risk and vulnerable populations | Children  MARPS  Disability |  | As above |  | - |
| **East Asia, Pacific** |  |  |  |  |  |  |  |  |
| Cambodia  CAM-708-G11-H | Indirect | Nutritional support to OVC households,  Transport for to hospital for PLWHA for TB x-ray | Protect, care and support OVC and families affected by HIV and AIDS | OVC  Families | 12 175 | $1 683 442 | $69 | 4% |
| Mongolia  MON-708-G05-H | Enterprise | Life skills training through harm reduction programme | Strengthening rehabilitation | IDUs | 633 | $40 838 | $32 | 0.01% |
|  |  |  |  |  |  |  |  |  |

IDUs = Injecting Drug Users; IGA = Income Generating Activities; MARPS = Most At Risk Populations; MSM = Men Who Have Sex With Men; PLWHA = People Living with HIV and AIDS; SW = Sex Workers
