# Supplementary material for: Economic Support to Patients in HIV and TB Grants in Rounds 7 and 10 from the Global Fund to Fight AIDS, Tuberculosis and Malaria
Source: PLoS One. 2014 Jan 28;9(1):e86225. doi: 10.1371/journal.pone.0086225 (PMC3904874; doi:10.1371/journal.pone.0086225)
Supplement: Table S2 — Round 7 TB grants that included direct and indirect forms of economic support in Phase 1 (first two years). (DOCX) [file pone.0086225.s002.docx]

Table S2: Round 7 TB grants that included direct and indirect forms of economic support in Phase 1 (first two years)

| **Region / Country** | **Type of transfer** | **Description** | **Rationale** | **Target group** | **No of people, units** | **Total expended on Living Support** | **$US/**  **pp/pa** | **Portion of SDA budget** | **Portion of total budget** |
| --- | --- | --- | --- | --- | --- | --- | --- | --- | --- |
| **East Africa, Indian Ocean** |  |  |  |  |  |  |  |  |  |
| Burundi  BRN-708-G06-T | Indirect | Nutritional support to MDR patients during full phase of hospitalization, hygiene parcles | Adherence to treatment | MDR patients | 61 | $2 873 | $23 | 57% | 2.6% |
| **Southern Africa** |  |  |  |  |  |  |  |  |  |
| Malawi  MLW-708-G06-T | Indirect | Food supplements to patients on MDR-TB treatment | Better adherence to treatment | MDR patients | Budget not allocated | | | | |
| **West, Central Africa** |  |  |  |  |  |  |  |  |  |
| Liberia  LBR-708-G06-T | Indirect | Nutritional support during intensive phase of treatment | Adherence to treatment | Patients |  | $378 132 |  | 24% | 3% |
| Senegal  SNG-708-G08-T | Indirect | Nutritional support to MDR patients | Adherence to treatment | MDR  patients | Budget not allocated | | | | |
| Sierra Leone  SLE-708-G06-T | Indirect | Food supplements to patients on MDR-TB treatment | Adherence to treatment | MDR patients | 10 000 | Budget not allocated | | | |
| **Latin America, Caribbean** |  |  |  |  |  |  |  |  |  |
| Cuba  CUB-708-G03-T | Enterprise | Training for PLWHA | Train PLWHA in TB/HIV issues | PLWHA | 632 | Budget not allocated | | | |
| Dominican Republic  DMR-708-G03-T | Enterprise | Train TB patients in technical areas | TB patients learn trades to facilitate their re-integration into the workforce and society | Patients |  | $99 274 | - | 14% | 0.7% |
|  | Indirect | Complimentary food, transport | Adherence to treatment, and transport for those who can’t afford it or with no easy access to health centres | Patients | Budget not allocated | | | | |
| Dominican Republic  DMR-708-G08-T | Indirect | Complimentary food, transport | Adherence to treatment, and transport for those who can’t afford it or with no easy access to health centres | Patients | Budget not allocated | | | | |
|  | Enterprise | Train TB patients in technical areas | TB patients learn trades to facilitate their re-integration into the workforce and society | Patients | Budget not allocated | | | | |
| **Middle East, North Africa** |  |  |  |  |  |  |  |  |  |
| Mali  MAL-708-G06-T | Indirect | Nutritional support to MDR patients | Hospitalization and nutritional support for 6 months to minimise the indirect costs of care | MDR patients | 325 | $7 537 | $11 | 0.03% | 0.07% |
| Somalia  SOM-708-G05-T | Indirect | Food support for TB patients | Better adherence to treatment | Patients |  | $312 000 | - | 10% | 0.7% |
| **Eastern Europe, Central Asia** |  |  |  |  |  |  |  |  |  |
| Azerbajan  AZE-708-G03-T | Indirect | Food parcels, transport, hygiene packages for patients on MDR treatment | Weekly food incentives for better adherence, transport to DOT centres | MDR patients | 192 | $237 917 | $619 | 6% | 1% |
|  | Enterprise | Vocational training | Training sessions for patients | MDR patients | Budget not allocated | | | | |
| **South, West Asia** |  |  |  |  |  |  |  |  |  |
| Iran  IRN-708-G03-T | Direct | Transport refund for DOTS visit | Adherence to treatment | Patients | 457 | Budget not allocated | | | |
| Nepal  NEP-708-G08-T | Direct | Educational support to children of TB-HIV co-infected people | Socio-economic rehabilitation | TB/HIV Co-Infected  Children |  | $136 160 | - | 8% | 0.9% |
|  | Indirect | Accomodation, transport and food support | Socio-economic rehabilitation and beds for poor MDR patients and their families or carers | MDR patients  Families | 465 | As above | - | - | - |
|  | Enterpris | Vocational training and IGA | Socio-economic rehabilitation | TB/HIV Co-Infected |  | As above | - | - | - |
| **East Asia, Pacific** |  |  |  |  |  |  |  |  |  |
| China  CHN-708-G11-T | Direct | Travel expenses, meal costs to patients on MDR treatment | Financial support during monthly visit to enable completion of 2 year and reduce loss to follow up | MDR patients | 70 | $347 665 | $2 838 | 135% | 14% |
| Lao  LAO-708-G10-T | Indirect | Nutritional support | Better adherence to treatment | Patients | 3 099 | $314 386 | $46 | 58% | 3% |
| Timor- Leste | Indirect | Supplementary feeding to MDR patients | Better adherence to treatment | MDR patients |  | $868 | - | 0.4% | 0.03% |
|  |  |  |  |  |  |  |  |  |  |
